# Challenges with adherence to the ‘test, treat, and track’ malaria case management guideline among prescribers in Ghana

**DOI:** 10.1186/s12936-022-04365-6

**Published:** 2022-11-15

**Authors:** Augusta Soninour Kolekang, Yaw Afrane, Stephen Apanga, Dejan Zurovac, Anthony Kwarteng, Samuel Afari-Asiedu, Kwaku Poku Asante, Anthony Danso-Appiah

**Affiliations:** 1 Department of Epidemiology, Biostatistics and Disease Control, School of Public Health, University for Development Studies, Tamale, Ghana; 2 Department of Medical Microbiology, College of Health Sciences, University of Ghana, Korle Bu, Accra, Ghana; 3 Department of Community Health and Preventive Medicine, School of Medicine, University for Development Studies, Tamale, Ghana; 4 KEMRI/Wellcome Trust Research Programme, Nairobi, Kenya; 5 Research and Development Division, Ghana Health Service, Greater Accra Region, Accra, Ghana; 6 Kintampo Health Research Centre, Research and Development Division, Ghana Health Service, Kintampo North Municipality, Bono East Region, Techiman, Ghana; 7 Department of Epidemiology and Disease Control, School of Public Health, University of Ghana, Legon, Accra, Ghana

**Keywords:** Adherence, Malaria, Test and treat, Prescribers, Challenges, And Ghana

## Abstract

**Background:**

Despite several efforts at addressing the barriers to adherence to the WHO-supported test, treat and track (T3) malaria case management guideline in Ghana, adherence remains a challenge. This study explored the challenges of prescribers regarding adherence to the T3 guideline.

**Methods:**

This was an explorative study using key informant interviews amongst prescribers comprising medical doctors, physician assistants, nurses and a health extension worker from 16 health facilities in six districts in Ghana. The data was analysed using Nvivo 10 and organized into thematic areas.

**Results:**

Prescribers lauded the guideline on testing and treatment as it ensures the quality of malaria case management, but irregular supply of malaria rapid diagnostic test kits (RDT), mistrust of laboratory tests, and the reluctance of prescribers to change from presumptive treatment were key barriers to testing. Patients with malaria test negative results if not treated, revisiting the facility with severe malaria, the experience of prescribers, lack of regular training and supervision for old and new staff and the inability of prescribers to investigate non-malaria fever hindered adherence to results-based treatment.

**Conclusion:**

As malaria remains a significant cause of morbidity and mortality in Ghana, this study provides insights on gaps in adherence to the testing and treatment of malaria. While the diagnostic capacity for malaria case management is a challenge, the lack of training resulting in the inability of some prescribers to investigate non-malaria fever hinders adherence to the malaria case management guideline. Therefore, there is a need to train new prescribers, laboratory personnel, and other staff involved in malaria diagnosis and treatment on the malaria case management guideline before they assume duty. Equipping laboratory personnel and prescribers with the knowledge to investigate non-malaria fevers could improve adherence to the guideline for improved patient care.

## Background

Five million deaths in low and middle-income countries were attributed to poor health care services in 2016 [1]. Prompt and accurate diagnosis and treatment of malaria are essential to reduce severe disease, complications, and the emergence of drug resistance [2, 3]. Accurate diagnosis and treatment will also reduce the cost of malaria case management and facilitate malaria control and elimination efforts. These have been difficult to achieve and, therefore, the World Health Organization (WHO) strategies and guideline for malaria control and case management have changed several times over the years [4, 5]. The Test, Treat, and Track (T3) policy initiated in 2012 recommends universal testing of all suspected malaria cases regardless of age and endemicity, treatment of positive test cases with quality assured anti-malarial drugs and tracking of all confirmed and treated patients [4].

Despite widespread adoption of the WHO guideline, adherence to the guideline by practitioners remains a challenge [6]. As of 2015, available data suggested that practitioners do not routinely request parasitological confirmation even when logistics are available [7, 8], and patients who present with malaria-related symptoms but test negative for malaria still get treated with anti-malarials [8] although non-treatment of negative cases is safe [9, 10]. Treating test-negative cases has serious implications as this could lead to misdiagnosis of cases with similar clinical presentation as malaria and consequently, inappropriate and delayed treatment [9].

While the availability of diagnostic and treatment logistics could influence adherence to the T3 guideline [11], the perceptions of prescribers and issues related to the performance of the diagnostic tests have been cited as major reasons for non-adherence to the guideline [12]. Some prescribers have expressed mistrust in laboratory results [13, 14], while others have reported that patients with negative results who do not get treated become positive in a few days [15]. Although sensitization of prescribers has been advocated as key to addressing these challenges, evidence is inconsistent on the effect of training and supervision on adherence to the guideline [16–19].

Malaria remains an important cause of morbidity and mortality in Ghana. It accounted for 34.5% of outpatient department (OPD) cases and 21.8% of all admissions in 2018 [20]. Malaria prevalence among children under-five years in Ghana was 14% in 2019 [21].

Ghana is among the malaria-endemic countries implementing the WHO T3 policy. The National Malaria Case Management Guideline was revised in 2014 to include mandatory testing of all suspected malaria patients across all age groups and treatment of uncomplicated malaria cases with one of the three recommended artemisinin-based combinations, namely artesunate-amodiaquine (AS-AQ), artemether-lumefantrine (A-L), and dihydroartemisinin-piperaquine (DHAP) [20, 22]. The current guideline was revised in 2020 with continuous emphasis on testing before treatment and adherence to test results [20]. To ensure adherence to the guideline, the Ghana Health Service (GHS) trained prescribers on the revised guideline on testing and treatment and improved the supply of logistics for malaria diagnosis and treatment [22].

However, adherence remains suboptimal. For example, according to the 2016 and 2019 Ghana Malaria Indicator Surveys (MISs), children under-five years with fever who had blood draw for the investigation of malaria declined from 34% in 2014 to 30% in 2016 but increase to 34% again in 2019 [21, 23]. Additionally, presumptive treatment of over 41% has been reported in Ghana [8, 19, 24]. Over 21% treatment of patients with malaria test negative results has also been reported in Ghana [19]. This study sought to explore challenges prescribers in Ghana face with adhering to the T3 guideline, detect further gaps influencing practices and local solutions to address challenges. This can help inform the implementation of the T3 strategy for prompt, accurate diagnosis and treatment of malaria cases that report to health facilities.

## Methods

### Study area and settings

**Fig. 1:**
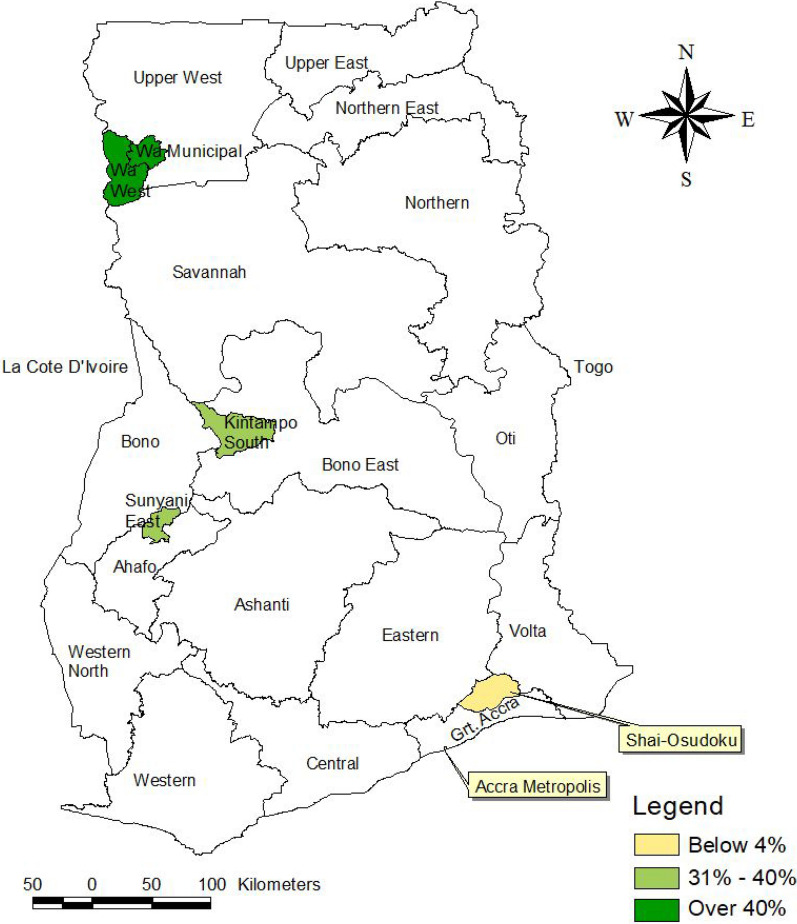
Map of study districts showing malaria prevalence among children 6–5 months in 2011

This study was conducted in six districts in three regions in Ghana, namely, the Greater Accra, the Upper West, and previously Brong Ahafo (now Bono East and Bono) Regions (Fig. 1). The districts were Wa Municipal, Wa West, Kintampo South, Sunyani East, Accra Metropolitan, and Shai-Osudoku. These districts were purposely selected to represent rural–urban settings and reflect differences in malaria prevalence and resource distribution in the country. The Wa Municipal and Wa West Districts are located in the Northern belt with an average malaria prevalence of over 40%; Kintampo South and Sunyani East Districts are in the middle belt with an average prevalence of malaria between 31% and 40%; while Accra Metropolitan and Shai-Osudoku Districts are in southern Ghana with an average prevalence of 4% according to the 2011 Multiple Indicator Cluster Survey [25]. The Wa West, Kintampo South, and Shai-Osudoku Districts represent rural districts, while Wa Municipal, Sunyani East, and Accra Metropolis represent urban districts.

Malaria cases are managed at all levels of the healthcare delivery system in Ghana which comprises of teaching hospitals, regional hospitals, district hospitals, polyclinics, clinics, health centres, maternity homes, Community Health and Planning Services (CHPS) compounds and licenced over-the-counter medicines shops [20, 22]. Apart from differences in the levels of care, healthcare facilities are also classified by ownership, and there are public, private, and faith-based owned facilities. Microscopy for blood film analysis which is the gold standard for testing malaria is performed mainly at teaching, regional and district hospitals; polyclinics; and clinics, whilst health centres; maternity homes; and CHPS compounds tend to rely mainly on rapid diagnostic tests (RDT) due to lack of human resources and logistics for microscopy.

### Study design and sampling

This was an explorative study conducted from October to December 2016. Key informant interviews (KIIs) were conducted among prescribers working in private, public, and faith-based health facilities across all levels of the health delivery system in the study area. Qualitative research seeks an in-depth opinion or knowledge of respondents with regards to a subject matter [26]. The health facilities were selected purposefully to reflect the different levels of health facilities and included both private and public facilities. Participants were also purposively selected in consultation with the head or in-charge of the outpatient department of the health facilities, to include prescribers who had worked as prescribers for at least six months. Background information on the facilities and prescribers was also collected. The interviews started in the Kintampo South District and ended in the Sha-Osudoku District.

### Study participants

Sixteen KIIs were conducted among prescribers comprising medical doctors, physician assistants, nurses, and a health extension worker. One respondent was taken from each facility. The research team co-designed the KII guide and piloted it with three respondents (2 nurses and 1 physician assistant) for its appropriateness. Based on the responses from the pilot study, the KII data collection guide was revised by the study team.

### Data collection

In-depth interview tools were used to examine barriers to testing suspected malaria cases and results-based treatment of confirmed uncomplicated malaria. Selected participants were informed about the purpose, procedures, and anonymity of the interviews, which were conducted face-to-face in English in the health facilities when attendance was very low to avoid interruption in health services delivery. The interviews were audio-recorded and conducted by research assistants who were trained on the study protocol and KII guide. Each interview session lasted for less than 45 minutes and was brought to an end when the moderator had exhausted all questions on the data collection guide and other emerging issues. On the background characteristic of the facilities, information on the availability of the Ghana Malaria Treatment Guideline, RDTs, diagnostic laboratory, and artemisinin-based combination therapy (ACT) were collected through observation (Appendix A). The period of the data collection was October to December 2016.

### Data management and analysis

The audio recordings of the interviews were transcribed verbatim by one of the trained data collectors with expertise in audio transcription. Transcripts were then checked for completeness and accuracy by vetting them to match the audio recordings by ASK and SAA, who also familiarized themselves with the data to gain a broad understanding of the content while also taking notes of important ideas. Transcripts were subsequently imported into Nvivo10 for coding where a priori themes were developed around barriers to testing of suspected uncomplicated malaria and results-based treatment of confirmed uncomplicated malaria. This was followed by open line-by-line coding and segment-by-segment coding while writing memos and looking for patterns. New themes and sub-themes that emerged during the coding process were discussed and included in the coding frame. This was followed by interpretive analysis as the results were presented in a narrative with quotes to support the findings.

## Results

### Characteristics of the health facilities and respondents

Out of the sixteen (16) health facilities, four (4) were district hospitals, four (4) were health centres, and three (3) were CHPS compounds. The study respondents were primarily nurses (n = 7) and medical doctors (n = 5) (Table 1).

**Table 1:** Characteristics of facilities included in the study and personnel interviewed

	Name of facility	Region	District	Type of facility	Prescribers (cadre)	Training on T3 guideline	Years of prescribing experience	Availability of a laboratory	RDT	Availability of T3 guideline	Availability of ACTs on day of survey		
											AS-AQ	A-L	DHAP
1	Charia Health Centre	Upper West	Wa Municipal	Health centre	Trained nurse	Yes	1	No	Yes	Yes	Yes	Yes	No
2	Domawa CHPS compound	Upper West	Wa West	CHPS compound	Health extension worker	Yes	2	No	No	Yes	Yes	Yes	No
3	Poyentanga Health Centre	Upper West	Wa West	Health centre	Trained nurse	Yes	5	No	No	Yes	Yes	Yes	No
4	Dangme West Hospital Complex	Greater Accra	Accra Metropolitan	Hospital	Medical doctor	Yes	14	Yes	Yes	Yes	No	Yes	No
5	Anyima Health Centre	Brong Ahafo	Kintampo South	Health centre	Trained nurse	Yes	Missing	No	Yes	No	Yes	Yes	No
6	Upper West Regional Hospital	Upper West	Wa Municipal	Hospital	Trained nurse	No	5	Yes	Yes	No	Yes	Yes	No
7	Adabraka Polyclinic	Greater Accra	Accra Metropolitan	Polyclinic	Medical doctor	Yes	8	Yes	Yes	No	No	Yes	Yes
8	Community Hospital, Shukura	Greater Accra	Accra Metropolitan	Hospital	Medical doctor	No	6	Yes	Missing	Yes	Yes	Yes	Yes
9	Osudoku Health Centre	Greater Accra	Shai-Osudoku District	Health centre	Physician assistant	Yes	3	Yes	Yes	Yes	Yes	Yes	No
10	Saint Andrews Clinic	Greater Accra	Shai-Osudoku District	Clinic	Physician assistant	Yes	3	Yes	Yes	Yes	No	Yes	No
11	Osuwem CHPS compound	Greater Accra	Dangme East	CHPS compound	Trained nurse	Yes	3	No	Yes	Yes	Yes	Yes	No
12	Kintampo South Hospital	Brong Ahafo	Kintampo South	Hospital	Medical doctor	Yes	13	Yes	Yes	No	Yes	Yes	No
13	Aspire Clinic, Sunyani	Brong Ahafo	Sunyani East	Clinic	Medical doctor	No	34	Yes	Yes	Yes	Yes	Yes	No
14	Islamic Hospital Wa	Upper West	Wa Municipal	Hospital	Trained nurse	No	20	Yes	Yes	Yes	No	Yes	No
15	Krabonso CHPS compound	Brong Ahafo	Kintampo South	CHPS compound	Trained nurse	Yes	2	No	No	Yes	Yes	Yes	No
16	Sunyani Polytechnic clinic	Brong Ahafo	Sunyani East	Clinic	Physician assistant	Yes	2	Yes	Yes	Yes	No	Yes	No

*AS-AQ* artesunate-amodiaquine, *A-L* artemether-lumefantrine, *DHAP* dihydroartemisinin-piperaquine

### Thematic areas

The main thematic areas identified are presented in three sections comprising of barriers to testing and results-based treatment, challenges to results-based treatment, and suggestions to improve adherence to recommendations in the malaria treatment guideline. The major themes of barriers to testing and results-based treatment included lack of laboratory and diagnostic facilities, erratic supply of RDTs, absence of laboratory technicians, delay and time-wasting, discordant results from RDT and laboratory, and the reluctance of prescribers to change. The major themes of challenges to results-based treatment included experience, lack of regular training and supervision, patients revisiting the facility with severe malaria when not treated, and lack of training for some categories of staff.

### Barriers to testing

While some respondents indicated the total adherence of their facilities, thus treating malaria based on laboratory confirmation, others sometimes treat malaria without laboratory confirmation. The following reasons accounted for this behaviour:

#### Inadequate laboratory facilities and rapid diagnostic tests

All CHPS compounds and most health centres in Ghana do not have laboratories to diagnose malaria necessitating the introduction of rapid diagnostic tests at these levels. At health centres without laboratories, RDT kits are sometimes unavailable. This finding is corroborated in the excerpt below;

*"We don't have the facilities (diagnostic tools) for the testing. So, if you run out of RDTs and your facility has no laboratory services or is far away from facilities that run laboratory services, then you can treat without laboratory confirmation depending on how the client may be suffering"* (KII with respondent A).

#### Erratic supply of RDTs

CHPS compounds largely rely on RDTs to confirm malaria before treatment, but the inconsistency in the supply of these kits leads to shortages in the health facilities. Therefore, prescribers are left with no option but to rely on clinical symptoms in the treatment of malaria when they run out of RDTs. This finding is encapsulated in responses from some key informants below.

One key informant said that;

*"At our level (CHPS compound), we only have access to RDTs, but in the case where there are no RDTs, and a patient presents with clinical symptoms of malaria, we treat once we don't have the RDTs"* (KII with respondent B).

Another respondent had this to say:

*"Well, we treat malaria without laboratory confirmation because there is no regular supply of RDTs and then secondly, because there is no laboratory that is closer to us. So, we just treat with the signs and symptoms the person presents"* (KII with respondent C).

#### Absence of laboratory technicians

To obtain an accurate malaria microscopy result requires a well-trained laboratory personnel. However, technicians are sometimes not at post due to their limited numbers, hindering malaria diagnosis before treatment.

A respondent had this to say concerning the absence of laboratory technicians:

*"Mostly we do the laboratory test before we treat, but sometimes when the patients come, and maybe as at the time they came, our lab technicians are not available, and the clinical presentation is pointing to malaria, we start treatment and then later when they come, we do the lab test."* (KII with respondent D).

#### Delay and time-wasting

The perceived delay by patients of the laboratory confirmation process for malaria emerged as one of the reasons prescribers treat malaria without laboratory confirmation. This point was made by a key informant who said that;

*"They (patients) think it is time-wasting. So when they come, and we just look at the signs and symptoms, and we treat, it will be faster for them. These are some of the things that make them treat without confirming"* (KII with respondent E).

Similarly, some health workers substantiated the views of patients with regard to the delay of the laboratory confirmation process for malaria. However, the delay was attributed to inadequate microscopes to run the test for queuing patients who are in some form of discomfort.

One respondent had this to say about delay and time-wasting:

*"Our challenge basically is getting the microscopy done quickly because we have just one microscope, and then sometimes we have as many as ten patients waiting in a queue. The waiting time becomes an issue because the patient is clinically not too well, so you would want to start something"* (KII respondent F).

#### Discordant results from RDT and microscopy

It was also revealed that some prescribers treat malaria without laboratory confirmation because of previous incidence of inconsistency in RDT and laboratory results. According to some respondents, there have been times that the RDT kit indicated negative malaria results but turned out to be positive after microscopy from the laboratory. Their argument is that RDTs could give false-negative results and as such, there is no need for laboratory confirmation when clinical symptoms of malaria are apparent.

A key informant intimated that;

*"…. our first line is usually the rapid diagnostic test, but then we noticed that sometimes we get some false-negatives from that, and when we send them for microscopy, we get parasitaemia levels of more than a thousand parasites per field. So, we start to treat malaria based on clinical symptoms because sometimes we can't wait for the microscopy to come out"* (KII with respondent G).

#### Mistrust of RDT and microscopy negative results

In as much as microscopy is proven to be specific and sensitive, some prescribers still do not trust the results, especially when the microscopy indicates a negative result but a patient is sick after other diseases have been ruled out.

This finding was succinctly explained by a key informant when he said;

*"Because sometimes we don't trust that the microscopy is right or maybe if they use the RDT, we don't trust it because maybe physically, the patient looks very ill. You've done other tests, and everything looks fine, so we still treat the malaria."* (KII with respondent H).

The decision of some prescribers to treat malaria without RDT or microscopy confirmation is informed by previous experience where prescribers treated patients for malaria though laboratory test was negative, as captured in the response below;

*"Sometimes, you will see it clearly with the signs and symptoms; the patient will even tell you it is malaria before the prescriber sees it. Most of the time, even though it is negative, based on the signs and symptoms, if they treat it, it works. So that is why I treat malaria with signs and symptoms even if the laboratory result is negative. Previously, I have treated it, and I have seen that it worked. The next one, I will go ahead to treat it again even if the laboratory test proves it negative"* (KII with respondent F).

#### Reluctance of prescribers to change

Some respondents mentioned the inability of prescribers to adopt the new malaria treatment guideline, which enjoins prescribers to test before treating malaria, as another reason prescribers treat malaria without laboratory confirmation. This finding is highlighted in a response by a respondent as;

*"……some of us (prescribers) are not availing ourselves to change. In most facilities, we are used to treating malaria without laboratory confirmation because we know that malaria presents these kinds of symptoms"* (KII with respondent I).

Closely linked to this finding was that some level of ambiguity compounds the inability of some prescribers to change with the malaria treatment guideline. A respondent has this to say in support of this finding.

"*Even in one of the guideline, they stated that if a child is convulsing, you treat, but then in the same guideline, they said don't treat. So that kind of ambiguity is also something that like sort of makes it difficult for the people to really, really change*" (KII with respondent I).

### Respondents’ reported challenges with results-based treatment

The challenges with results-based treatment are presented under the following themes:

#### Experience

Experience from medical practice was one of the reasons prescribers treat malaria after results have been confirmed at the laboratory as negative. There were three related findings on experience;

1) Some patients, especially adults, have never tested positive for malaria yet recover when treated for malaria based on the signs and symptoms as elaborated by one respondent below.

*"Yes, yes. You know, if you have been a prescriber for some time, you will get that experience that some people, for one reason or the other, have never tested positive for malaria. Meanwhile, they have the signs and symptoms. And when you treat them for malaria, they are fine"* (KII with respondent A).

Another respondent had this to say about his experience.

*"This is my personal experience; when I have malaria with classical signs and symptoms, I will do the test, and it will be negative, but when I take the anti-malarial, I'm okay"* (KII with respondent D).

Furthermore, in support of treating patients who have obvious signs and symptoms of malaria although may test negative, one respondent indicated that;

*"We also know that sometimes the parasites are hiding in the liver, so when you do the test, you might not see them, it might not be positive"* (KII with respondent H).

2) It also emerged that some prescribers continue to treat some patients for malaria regardless of negative results from the laboratory because such patients have revisited the facility in a worsened condition after being treated for a different illness and discharged.

A respondent had this to say in connection with this.

*"…… some of them too when they come with malaria confirmed negative results, and you treat them for a different condition, and they go, they still come back after treatment which shows that they were really having malaria, but the laboratory was not able to detect that, or maybe the laboratory was faulty or the reagents"* (KII with respondent A).

3. Also, some respondents alluded that prescribers treat malaria after other disease conditions have been treated, but patients are still ill.

*"Most of the time, when it is negative, we don't treat. We look for other conditions. But where there might be other conditions treated and still the patient is not getting better, and the clinical signs and symptoms are clear, then irrespective of the lab result, we go ahead and treat, and most of the time when you treat, they are fine"* (KII with respondent D).

#### Lack of regular training and supervision

Some respondents noted the lack of training and supervision as one of the reasons why some prescribers treat malaria cases confirmed as negative by the laboratory. In this regard, it was revealed that new staff are not trained on the malaria treatment guideline upon coming on duty as replacement of trained staff who are either going on transfer or furthering their education.

Throwing more light on the lack of regular training and supervision, one key informant had this to say;

*"Yeah, at times we do that because there is no training and supervision; there are no funds to organize the training and also come for supervision. Another factor is that when trained staff are transferred to a different place or go to school, some of the new staff who come are not trained. That's another factor"* (KII with respondent C).

On ensuring that patients with negative malaria test results are not treated for malaria, some respondents indicated that their facilities do not have a challenge in this regard.

*"Virtually, we don't have much challenge because like I said if it is negative, we don't treat unless we treat other conditions and still we are not getting any positive result or the patient is still sick and then comes back for review”* (KII with respondent D).

This notwithstanding, other respondents raised the following challenges:

#### Patient revisiting the facility with severe malaria when not treated

Prescribers are not able to let go without treating patients who test negative for malaria because they are likely to return to the facility with severe malaria. Also, it appears prescribers treat patients who test negative for malaria because the patient may have to bear the cost of care when they revisit the facility within a specific number of days, as indicated by the National Health Insurance Authority. This finding was captured in the response below;

*"It will be a problem for some other clients because it means within a month or even less, people are going to revisit the facility with the same complaints or even worse because the lab confirmed that they do not have malaria and so you failed to treat for malaria. They are likely to come back. Their coming back will cost you, especially with the capitation being introduced by the National Health Insurance Scheme"* (KII with respondent A).

Another respondent also shared his/her experience with regard to patients revisiting the health facility with severe malaria when not treated even though the patient may have tested negative for malaria as;

*"The challenge here is that when one is tested for malaria, and it is negative, based on the protocol or the guideline we are not allowed to treat. So I have seen that at times one will be having the malaria parasites in him/her, but once the test is negative and he/she is not treated for malaria, subsequently the person comes back with huge numbers of parasites in his/her blood because they are harboring the parasites in them. These patients come back with a severe life-threatening condition, and later they test positive for malaria"* (KII with respondent B).

#### Lack of training for other staff

It also emerged that in facilities where health staff other than trained prescribers are involved in the treatment of malaria, they are likely to treat malaria based on clinical symptoms because they are not trained to diagnose other possible causes of diseases if patients test negative for malaria. As encapsulated in the response below, particular reference is made to those who are most often involved in treating malaria in the health centres and CHPS compounds.

*"The challenge especially now is with a professional group. They should be trained to be able to elicit other causes of disease conditions. Because they are not able to elicit other conditions, they are compelled to treat for malaria"* (KII with respondent I).

### Suggestions to improve adherence to the malaria treatment guideline

Respondents unanimously upheld the relevance of the recommendation in the malaria treatment guideline that all parasitologically confirmed negative malaria should not be treated with an anti-malarial.

The following responses were suggestions from some respondents as to how to improve on adherence to the recommended malaria treatment guideline:

*"Once the laboratory says the cases are not confirmed, you don't go ahead and treat them. But you can do further investigations. You can request the laboratory test from another facility to find out because there are instances whereby we run blood film (BF) here and is negative. The cases go outside and then is positive"* (KII with respondent J).

*"My thought is that it shouldn't be treated when it's negative because there are a lot of conditions with similar signs and symptoms. So, when you test and is negative, you just have to investigate more, perhaps do another test to be sure that what you are diagnosing is the real thing"* (KII with respondent C).

While respondents generally upheld that the guideline presents an opportunity for further medical examination/diagnosis when test results are negative for malaria, others alluded that prescribers may have to treat malaria after all possible causes are ruled out.

*"This is a challenging question, though, but then it all boils down to the one seeing the case and then the stage of the infection. If all other possible causes are ruled out, and there are signs and symptoms of malaria, then you can go ahead and treat malaria. That's what I can just tell you"* (KII with respondent J).

*"If you are really convinced with the signs and symptoms in addition to the negative laboratory results, I don't think there is the need for you to still go ahead to treat with an anti-malarial. However, if the laboratory result has confirmed malaria negative but the signs and symptoms the person is presenting are suspicious of malaria, I think you should treat because if you don't do that, they will go and come back"* (KII with respondent A).

## Discussion

The study explored the perceptions and challenges of prescribers on adherence to the Ghana Malaria Case Management Guideline and solutions to improving adherence. Prescribers generally perceived the recommendations as good. However, some human and logistic challenges were expressed. Lack of, and inadequate malaria diagnostic tools (microscopy and rapid diagnostic test) and human resources to conduct microscopy hinder adherence to the malaria case management guideline. This was prominent in the interviews as documented in the literature [8, 27]. Rapid diagnostic test kits, which are mainly reserved for testing malaria in rural and remote health facilities such as the CHPS compounds, were either found to be in short or erratic supply.

If prescribers comply with the guideline consistently, it will result in delays at the OPD because of inadequate personnel and logistics, especially microscopes for diagnosis. This means patients will not get their laboratory results promptly to complete their OPD procedures. Prescribers echoed this as a barrier to adherence to the guideline. Delay in receiving care at a health facility could worsen the condition of patients, prevent future attendance or generate poor health-seeking behavior among patients. Therefore, efforts should be made to ensure adequate logistics and human resources to implement the guideline effectively and efficiently, and motivate positive health-seeking behavior among patients. An innovation such as pre-consultation testing has been shown to increase adherence to testing and treatment and reduced the time mothers of children spend at the OPD [28]. Both prescribers and caregivers of children preferred the pre-consultation testing to the usual consultation practice before laboratory testing [28].

The lack of trust in negative results preventing some prescribers from relying on them for patient management is prevalent, especially results from RDT [12]. Some prescribers reported contradictory results of patients tested at different places. While this could result from differences in the sensitivity of microscopy and malaria rapid diagnostic tests (RDT), inadequately trained diagnostic staff could contribute to inconsistent results [23]. Additionally, the report of detection of *Plasmodium falciparum* with deleted Histidine-rich protein 2 (*hrp2*) and Histidine-rich protein 3 (*hrp3*) genes could contribute to the false-negative results obtained from RDTs [5]. More research will be needed to ascertain the prevalence of *P. falciparum* with deleted *hrp2* and *hrp3* genes to inform policy on the continuous use of the RDT. Proper training of diagnostic personnel on microscopy is required to ensure that patients are not given contradictory results. Community-based health planning services facilities without the capacity to perform microscopy could liaise with nearby healthcare facilities with the capacity for their microscopy testing.

While laboratory tests are crucial in the practice of evidence-based medicine and ensure that patients get the treatment based on the best available evidence, the influence of the experience of a prescriber on patient management cannot be overstated [29]. The combination of the two in the management of patients featured very much in the narratives of prescribers on why they treat patients with test negative results. Prescribers indicated that when the patient's clinical presentation is not consistent with the laboratory results, experience, rather than laboratory results, dominates in the treatment decisions made by the prescriber [30]. This could lead to missed diagnosis when further investigations to identify the cause of the fever are not done due to the similarity of symptoms of malaria with other conditions [9]. Therefore, equipping laboratories to investigate alternative causes of fever will be crucial in improving the quality of malaria case management. Some also indicated that from experience, some people, even when they habour the parasites, will never test positive, which accounts for their treatment of test-negative cases with anti-malarial. More evidence to show that relying on laboratory tests and withholding anti-malarial from malaria test-negative patients do not have negative consequences could convince prescribers to adhere to the guideline [10].

Still related to experience is the fear of test-negative patients not treated returning to facilities in a worse state [12]. While the impact of this could be minimal with comprehensive laboratory investigation, it could result in loss of trust of patients in the ability of prescribers [10]. This also has the potential to generate negative health-seeking behaviour among patients. Besides, the fact that some also indicated that some patients who test negative get better after such treatment with anti-malarial poses a challenge to adherence to the guideline.

Lastly, adherence to test negative results becomes challenging to prescribers if they cannot detect the cause of a patient's illness or have treated a patient for another condition, but the patient fails to recover. To this, some prescribers indicated that lack of training on alternative diagnoses for patients with negative test results, especially for nurses and newly recruited staff, limits the ability of nurses to explore other causes of a patient's ill-health. Challenges with alternative diagnoses have been documented [9]. The lack of diagnostic capacity of facilities to investigate other possible causes of fever in malaria test-negative patients could account for the non-adherence. This situation requires equipping prescribers with the knowledge to make alternative diagnoses and resourcing health facilities with the diagnostic logistics. Since malaria is a common cause of under-five morbidity and mortality, especially among children under-five years, the Ghana Health Service should ensure that all prescribers are trained on the guideline. This could be included in the training curricula for prescribers as well as incorporated into the orientation of new staff.

Although the prevalence of malaria among children under-five years has declined since the conduct of this study (21% in 2016 to 14% in 2019), it is still imperative for prescribers to adopt such changes in their practice [21]. Resistance to change as a reason for prescribers not adhering to the guideline hampers the quality of malaria case management and the overall malaria control agenda. Accurate testing and treatment will ensure accurate surveillance data for appropriate responses and also for the evaluation of malaria control interventions [4]. Reluctance to change has been documented in other studies and could be a consequence of inadequate information about the guideline [12]. Training and supervision will keep prescribers up-to-date on the guideline whenever the guideline is updated. This will avoid ambiguity with the previous guideline documented in this study and enable prescribers to adhere to the guideline.

### Strengths and limitation of the study

Prescribers included in this study comprised all the different cadre of prescribers in the country. In addition, prescribers from rural and urban centres and the different categories of facilities were also included. However, the qualitative nature of the study limits the generalizability of the study findings.

## Conclusion

While prescribers lauded that Ghana adopted the WHO test, treat and track recommendations on malaria case management, there are concerns with optimum adherence to the guidelines. Logistical challenges, mistrust in laboratory results and inadequate training of health workers hinder the T3 policy. Equipping laboratory personnel and prescribers with the knowledge to investigate non-malarial fever could improve adherence to the guideline for improved patient care.

## Data Availability

The datasets used in the current study are available from the corresponding author upon reasonable request.

## Appendix A

See Table 1
